# Transcriptome Analysis Reveals Genes and Pathways Associated with Drought Tolerance of Early Stages in Sweet Potato (*Ipomoea batatas* (L.) Lam.)

**DOI:** 10.3390/genes15070948

**Published:** 2024-07-19

**Authors:** Peng Cheng, Fanna Kong, Yang Han, Xiaoping Liu, Jiaping Xia

**Affiliations:** Key Laboratory of Crop Quality Improvement of Anhui Province, Crop Research Institute, Anhui Academy of Agricultural Sciences, Hefei 230001, China; cp208@126.com (P.C.); kongfanna2009@163.com (F.K.); minisectrl1@163.com (Y.H.); xiaopingliugs@163.com (X.L.)

**Keywords:** sweet potato, drought stress, transcriptome, RNA-seq, root, leaf

## Abstract

The yield of sweet potato [*Ipomoea batatas* (L.) Lam] can be easily threatened by drought stress. Typically, early stages like the seedling stage and tuber-root expansion stage are more vulnerable to drought stress. In this study, a highly drought-tolerant sweet potato cultivar “WanSu 63” was subjected to drought stress at both the seedling stage (15 days after transplanting, 15 DAT) and the tuber-root expansion stage (45 DAT). Twenty-four cDNA libraries were constructed from leaf segments and root tissues at 15 and 45 DAT for Next-Generation Sequencing. A total of 663, 063, and 218 clean reads were obtained and then aligned to the reference genome with a total mapped ratio greater than 82.73%. A sum of 7119, 8811, 5463, and 930 differentially expressed genes were identified from leaves in 15 days (L15), roots in 15 days (R15), leaves in 45 days (L45), and roots in 45 days (R45), respectively, in drought stress versus control. It was found that genes encoding heat shock proteins, sporamin, LEA protein dehydrin, ABA signaling pathway protein gene *NCED1*, as well as a group of receptor-like protein kinases genes were enriched in differentially expressed genes. ABA content was significantly higher in drought-treated tissues than in the control. The sweet potato biomass declined sharply to nearly one-quarter after drought stress. In conclusion, this study is the first to identify the differentially expressed drought-responsive genes and signaling pathways in the leaves and roots of sweet potato at the seedling and root expansion stages. The results provide potential resources for drought resistance breeding of sweet potato.

## 1. Introduction

Drought stress seriously affects the productivity of crops worldwide [1]. Due to climate fluctuations and irregular rainfall, crops are increasingly exposed to drought and high temperatures [2]. Drought stress affects ionic and osmotic homeostasis signaling pathways, detoxification response pathways, and pathways for growth regulation [3]. Drought stress first causes cellular water loss and then induces reactive oxygen species accumulation, followed by damage to cellular components such as membrane lipids, proteins, and nucleic acids, and metabolic dysfunction [4].

Sweet potato (*I. batatas* (L.) Lam.) is one of the most important root crops cultivated worldwide [5]. Because of its adaptability, high yield potential, and nutritional value, sweet potato has become an important food crop, particularly in developing countries [6]. This crop is often grown on ridges and is easily subjected to drought stress after long-term dry weather [7].

Transcriptomic and proteomic analyses have provided a new approach to understanding drought tolerance mechanisms. Proteomic analysis of four tomato species and transcriptomic analysis of *Sulla coronaria* (L.) have uncovered many pathways involved in drought stress response [8,9]. Lau et al. (2018) discovered 122 drought-responsive genes using RNA-Seq analysis [10]. Arisha et al. (2020) studied the purple-fleshed sweet potato “Xuzi-8” and identified many drought-responsive unigenes, many of which encode osmolyte biosynthesis enzymes, water channels, sugar and proline transporters, detoxification enzymes, chaperones, and late embryogenesis abundant (LEA) proteins [11]. Transcriptome analysis of seven sweet potato cultivars found that pathways involved in plant signal transduction, flavonoid biosynthesis, phenypropanoid biosynthesis, and isoquinoline alkaloid biosynthesis play important roles in regulating drought stress tolerance [12].

Previous studies have demonstrated that drought stress can reduce the root yield of sweet potato to varying degrees, and water scarcity in the early stages has a more significant impact on sweet potato yield than in the later stage [13]. To elucidate the drought tolerance mechanism in the early stages of sweet potato, in this study, transcriptomic analysis of roots and leaves from the drought-tolerant sweet potato cultivar “WanSu 63” was conducted on the Illumina NovoSeq6000 platform at 15 and 45 days after transplanting. These transcriptome sequencing data from hexaploid sweet potato under drought stress can provide a useful resource for sweet potato breeding and offer new candidate genes that constitute a valuable reference for the furtherance of research on the functional genomics of sweet potato.

## 2. Materials and Methods

### 2.1. Plant Growth Conditions

The yellow-fleshed sweet potato cultivar “WanSu 63” was used as the experimental material. Uniform healthy shoots of the top 25 cm were planted in each pot; the pots were 30 cm in diameter and 60 cm in height. One plant was planted per pot, and there were 30 pots for each treatment, with a total of 60 pots. Drip irrigation was used to precisely control the soil moisture throughout the entire growth stage. The soil moisture was measured using a TDR150 soil moisture meter (Spectrum technologies, Inc., Aurora, IL, USA), with 60 ± 5% soil water content as the normal treatment, and 30 ± 5% soil water content set as drought stress treatment. Samples were taken 15 and 45 days after drought stress (i.e., seedling stage and root expansion stage, respectively).

On days 15 and 45 after drought stress, the third, fourth, and fifth unfolded leaves from the main vine of representative individual plants were collected and then mixed as leaf samples. Fibrous roots (tubers) were also collected as root samples for RNA extraction, followed by transcriptome sequencing (8 samples with 3 biological replicates each).

At harvest, 10 representative individual plants from each treatment were selected. The number of tubers per plant, the fresh weight of tubers, the fresh weight of stems and vines, the length of vines, and the number of branches were measured.

### 2.2. Drought-Related Physiological Chemical Contents and Yield-Related Indexes

Samples were taken 15 and 45 days after transplanting. The proline content was quantified using a colorimetric assay kit (Catalog No. A107) sourced from Nanjing Jiancheng Bioengineering Institute. Furthermore, the enzymatic activities of superoxide dismutase, as well as total antioxidant capacity (T-AOC), were assessed using respective kits from the same institute (Catalog No. A001-1 and A015, respectively). The concentration of ABA we measured using a competitive enzyme-linked immunosorbent assay [14].

Sweet potato was grown on 12 June and harvested on 23 October, with a life span of 132 days. The yield-related traits of sweet potato including length of vine, tuberous root number, branching number, tuberous root weight, aboveground biomass, and total biomass were measured at harvest. The fresh tuberous roots were shaved into shreds and baked in an oven at 85 degrees Celsius for 48 h until a constant weight was reached, and the dry rate was calculated subsequently.

### 2.3. Total RNA Isolation and Library Construction

Total RNA was isolated using Trizol reagent (Invitrogen, Waltham, MA, USA) according to the manufacturer’s protocol. RNA concentration was measured using Qubit^®^ RNA Assay Kit in Qubit^®^ 2.0 Flurometer (Life Technologies, Carlsbad, CA, USA). RNA integrity was assessed using the RNA Nano 6000 Assay Kit of the Agilent Bioanalyzer 2100 system (Agilent Technologies, Santa Clara, CA, USA).

A total amount of 1.5 μg RNA per sample was used as input material for the RNA sample preparations. Sequencing libraries were generated using the NEBNext^®^ Ultra^TM^ RNA Library Prep Kit for Illumina^®^ (NEB, Ipswich, MA, USA) following the manufacturer’s recommendations, and index codes were added to attribute sequences to each sample. The concentration of the libraries was initially measured using Qubit^®^2.0 (Life Technologies, Carlsbad, CA, USA). The libraries were diluted to 1 ng/μL and an Agilent Bioanalyzer 2100 (Agilent, Santa Clara, CA, USA) was used to test the insert size of the libraries.

Next, we used the Illumina NovoSeq 6000 (Illumina, San Diego, CA, USA) sequencing platform to explore the transcriptome in leaf tissues (3rd, 4th, and 5th unfolded leaves collected as leaf samples) and root tissues 15 days and 45 days after transplanting, respectively.

### 2.4. Analysis of Differentially Expressed Genes

Raw reads were filtered using SOAPnuke [15] software (v2.1.0) to obtain clean reads; the main parameters were as follows: -lowQual = 20, -nRate = 0.005, -qualRate = 0.5, other parameters default. The clean reads were mapped to the *I. batatas* genome ([version 2019] on the website https://sweetpotao.com/download_genome.html, accessed on 10 June 2023) using HISAT2 (V2.1.0) software [16]. We mapped the reads to the merged transcriptome set using bowtie2 (V2.3.5) [17] and quantified the normalized expression level (FPKM) of each gene and transcript using RSEM (V1.3.1) [18].

Differentially expressed genes between sample groups were evaluated using DESeq2 (V1.22.2) [19]. The false discovery rate (FDR) was used to identify the threshold *p* values in multiple tests to compute the significance of the differences. Here, only genes with |log2(FoldChange)| ≥ 1 and FDR significance score (padj) < 0.01 were selected for subsequent analysis.

### 2.5. Gene Ontology Term Enrichment Analysis and KEGG Enrichment Analysis

Differentially expressed genes were compared against various databases for their functional annotations. We compared plant-specific sequences from the NCBI NR database and the SwissProt database using BLASTX (V2.6.0) with an e-value cut-off of 10^−5^. The best blast-hit based on bit-score was used for subsequent functional annotation. Gene Ontology annotation was performed based on the corresponding genes in NCBI and their GO annotations [20]. The database of this correspondence was obtained from https://ftp.ncbi.nlm.nih.gov/gene/DATA/gene2go.gz (accessed on 12 June 2023). KEGG pathway annotation was performed using BLASTx against the plant-specific sequences from the KEGG database [21]. GO and KEGG enrichment was performed using the hypergeometric test as implemented in the R phyper function.

## 3. Results

### 3.1. Physiological Responses of Tuberous Roots and Leaves under Drought Stress

**Figure 1:**
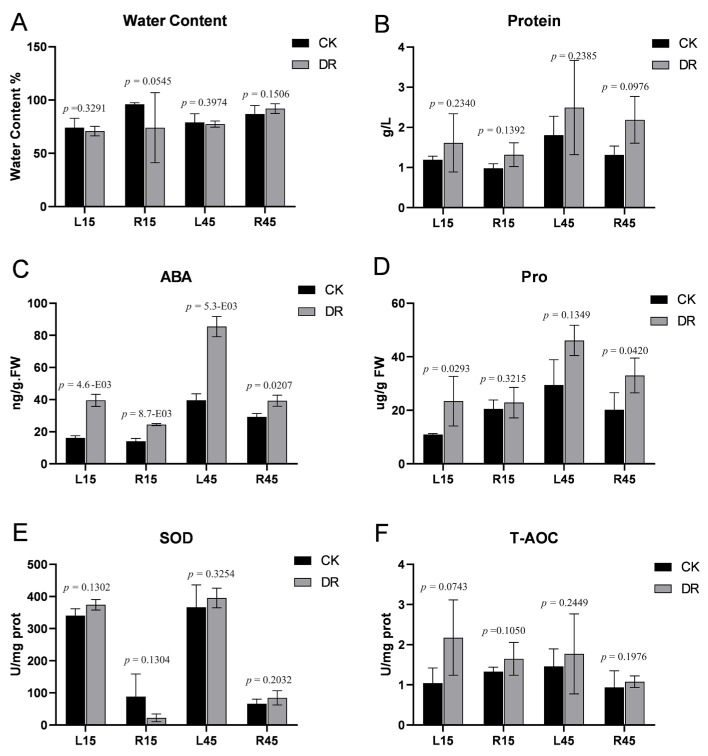
Biochemical analysis of sweet potato samples under drought stress. (**A**) Water content. (**B**) Total protein content. (**C**) Abscisic acid content. (**D**) Proline content. (**E**) Superoxide dismutase activity. (**F**) Content of total antioxidant capacity. CK: control; DR: drought stress treatment. Bars mean SD (*n* = 3). *p* represents the result of Student’s *t*-test.

Drought stress severely disturbs plant growth. To investigate the physiological response of sweet potato to drought, we measured the content of water, proline, ABA, and total protein. The results showed that under stress, water content slightly decreased in leaves from sweet potato 15 days after transplanting (L15), roots 15 days after transplanting (R15), and leaves from sweet potato 45 days after transplanting (L45) but was not affected in roots from sweet potato 45 days after transplanting (R45) between drought versus each control, respectively (Figure 1A). Total protein content increased after drought stress in sweet potato in all comparisons (Figure 1B). The ABA concentration significantly increased in all comparisons as expected, and was more pronounced in leaf tissue. (L15 and L45) (Figure 1C). We also measured some enzyme activities. After drought, the T-AOC (total antioxidant capacity) increased in all comparisons (Figure 1F). The content of proline also increased in all comparisons as expected. SOD enzyme activity increased in all tissues and was notably higher in leaf tissues (L15 and L45) compared to root tissues (R15 and R45) in drought stress versus control, respectively (Figure 1E).

The growth of sweet potato is severely inhibited under drought stress. The harvest-related traits were measured at 132 days after transplanting. Except that the tuberous root numbers slightly increased, the other traits including length of the main vein, branch number, total biomass, aboveground biomass, and tuberous root weight significantly decreased after drought stress (Supplemental Figure S2).

### 3.2. Transcriptome Sequencing, Quality Filtering, and Assembly

Twenty-four cDNA libraries were constructed, and 198.9 Gb of clean data was generated after adaptor sequences and low-quality reads were removed. The correlation heatmap showed that three biological replicates within each sample have the highest correlation (Figure S1). In all libraries, the Q30 ratio ranges from 91.89 to 94.77% and guanine–cytosine (GC) content ranges from 46% to 49% (Table 1). The ratio of genomic reads to clean reads was greater than 65% in R45 tissue and 70% in the other tissues, and total mapped reads ranged from 82% to 87%, which was sufficient for the next step of the analysis (Table S1).

### 3.3. Comparison of Differentially Expressed Genes among All Four Libraries between Drought Versus Corresponding Control Samples

The carotenoid-rich cultivar ‘Taizhong 6’ genome sequence (Version 2019) was used as the reference genome [22,23]. This version was re-sequenced using nanopore sequencing (Oxford Nanopore Technologies, Oxford, UK) to improve sweet potato genome assembly quality and is now the latest version [22]. Sweet potato cultivars with different drought tolerance performances showed distinct mechanisms for their response to drought stress [12].

As mentioned above, the level of gene expression in individual samples was calculated by FPKM, using the criteria of FDR < 0.05 and |log2 of fold change (FC)| >1. DEGs were generated in pairwise comparisons in different tissues at two developmental stages to observe the drought stress response, i.e., CK_L15-vs-DR_L15 (7119 DEGs in total, with 2906 up-regulated and 4213 down-regulated), CK_L45-vs-DR_L45 (5463 DEGs in total, with 1744 up-regulated and 3719 down-regulated), CK_R15-vs-DR_R15 (8811 DEGs in total, with 2996 up-regulated and 5815 down-regulated), and CK_R45-vs-DR_R45 (930 DEGs in total, with 445 up-regulated and 485 down-regulated) (Table S2). The results showed that as the sweet potato grew, the number of differentially expressed genes declined sharply, especially in root tissues. Compared with L15 tissues, DEGs in L45 tissues declined by 23%, whereas in R15 tissue, with 8811 DEGs, the number declined to 930 DEGs in R45 tissue, a decline of 89%. This indicates that drought tolerance increased as the sweet potato grew, and roots were more tolerant to drought stress than leaf tissues (Table 1).

**Figure 2:**
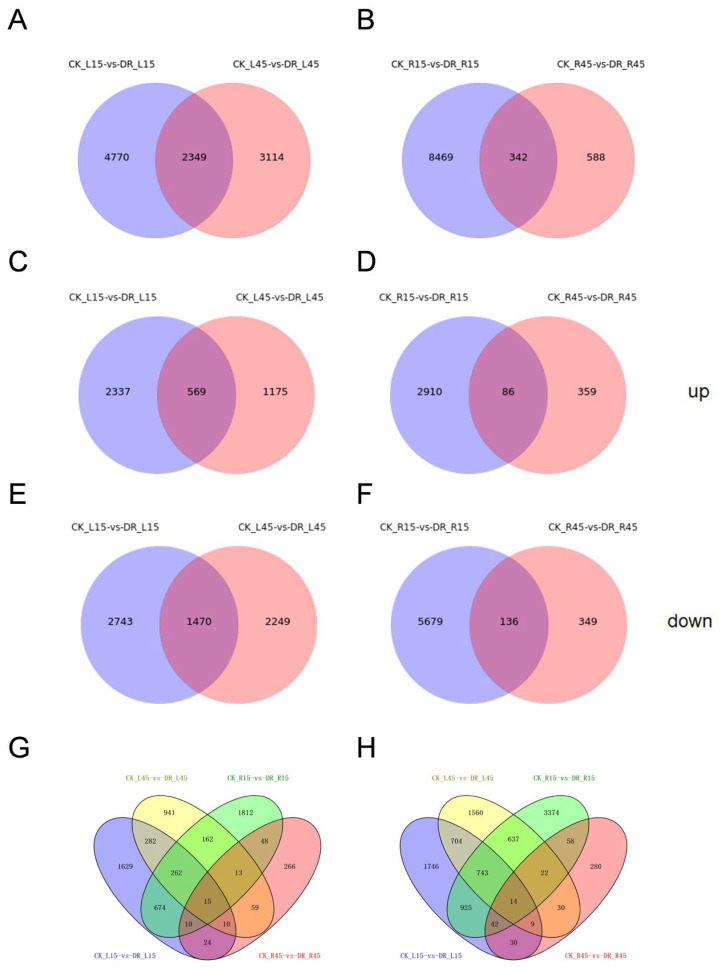
Venn diagram of DEGs. (**A**) DEGs in CK_L15-vs-DR_L15 versus CK_L45-vs-DR_L45. (**B**) DEGs in CK_R15-vs-DR_R15 versus CK_R45-vs-DR_R45. (**C**) Up-regulated DEGs in CK_L15-vs-DR_L15 versus CK_L45-vs-DR_L45. (**D**) Up-regulated DEGs in CK_R15-vs-DR_R15 versus CK_R45-vs-DR_R45. (**E**) Down-regulated DEGs in CK_L15-vs-DR_L15 versus CK_L45-vs-DR_L45. (**F**) Down-regulated DEGs in CK_R15-vs-DR_R15 versus CK_R45-vs-DR_R45. (**G**) Up-regulated DEGs in all four stages and tissues. (**H**) Down-regulated DEGs in all four stages and tissues.

The DEGs from root tissues differ from those of leaf tissues (Figure 2G,H, Supplemental Figure S2). There are only 15 commonly up-regulated and 14 commonly down-regulated DEGs in all four comparisons (Figure 2G,H). There are 2349 common DEGs between CK_L15-vs-DR_L15 and CK_L45-vs-DR_L45, of which 569 are up-regulated and 1470 are down-regulated (Figure 2A,C,E). There are also 256 common DEGs between CK_R15-vs-DR_R15 and CK_R45-vs-DR_R45, of which 342 are up-regulated and 86 down-regulated (Figure 2B,D,F).

**Figure 3:**
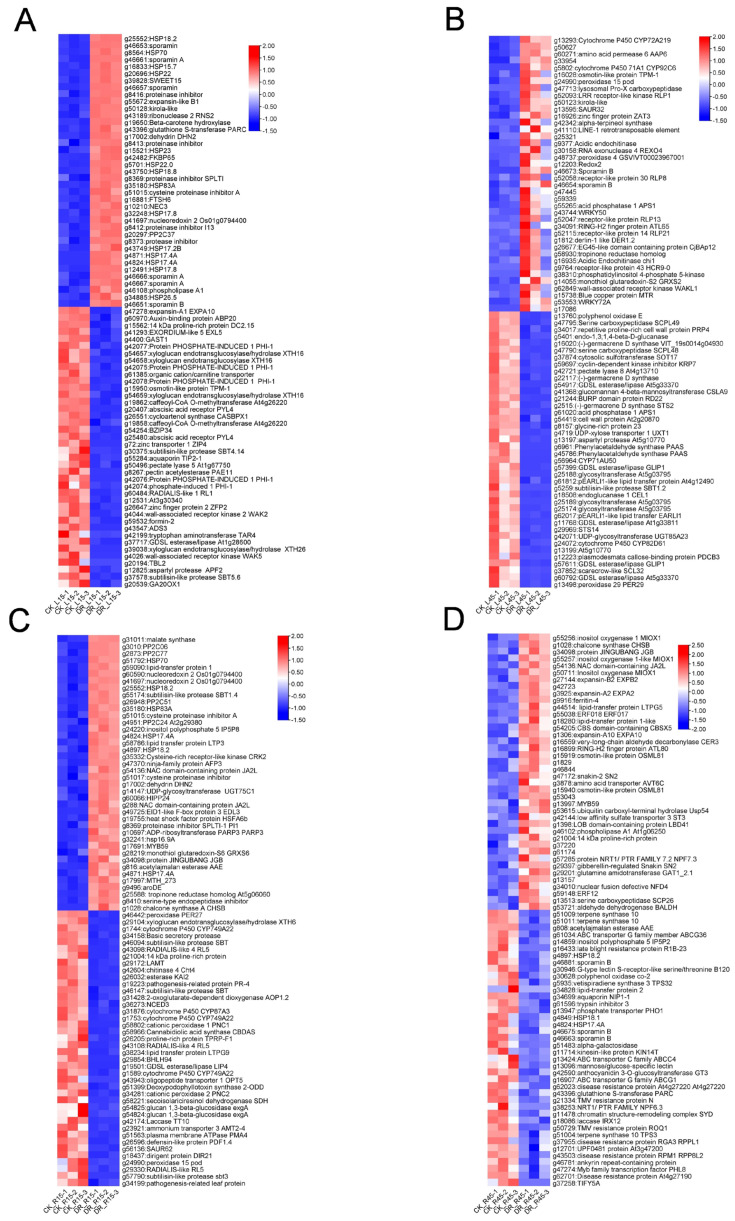
Most significant differentially expressed genes in all four stages and tissues. (**A**) DEGs in CK_L15-vs-DR_L15. (**B**) DEGs in CK_L45-vs-DR_L45. (**C**) DEGs in CK_R15-vs-DR_R15. (**D**) DEGs in CK_R45-vs-DR_R45. Red indicates up-regulated genes and blue indicates down-regulated genes. The genes were screened by *p*-value.

A group of genes encoding heat shock protein, sporamin, LEA protein, and genes involved in ABA Biosynthesis and Signaling Pathway were significantly enriched in DEGs (Figure 3). Genes involved in osmotic homeostasis, damage control and repair. In L15 stage, HSP encoding genes accounted for 35%(14 in 40) in top 40 up-regulated DEGs. The same trends occurred in R15 stage as well (Figure 3A,C). Several inositol oxygenase family gene members extremely enriched in R45 (Figure 3D).

### 3.4. Functional Annotation and Classification of DEGs

**Figure 4:**
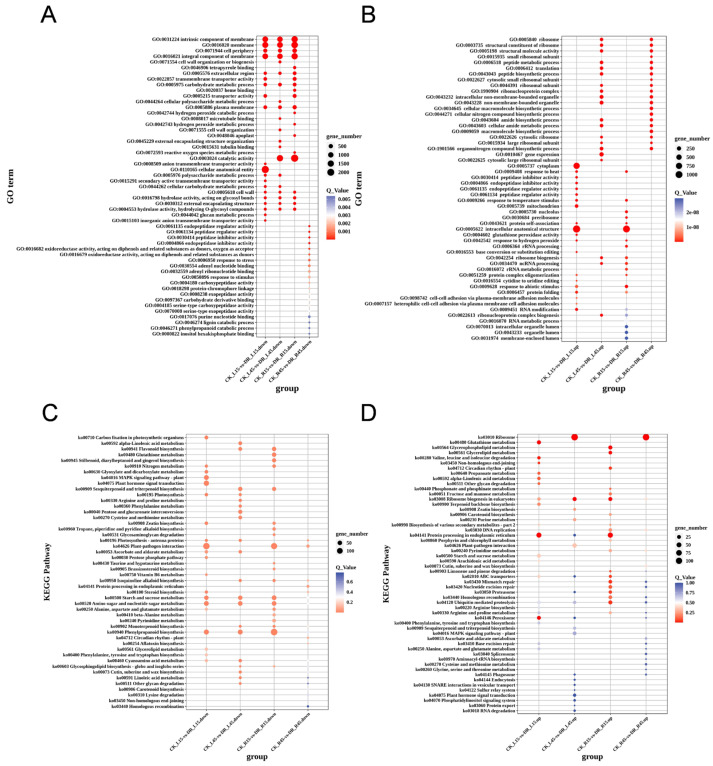
GO and KEGG enrichment in all four stages and tissues. (**A**) GO term enrichment of down-regulated DEGs. (**B**) GO term enrichment of up-regulated DEGs. (**C**) KEGG term enrichment of down-regulated DEGs. (**D**) KEGG term enrichment of up-regulated DEGs.

The GO (Gene Ontology) database is suitable for various species and can define and describe genes and proteins. Genes can be categorized into three main GO categories: BP (biological process), CC (cellular component), and MF (molecular function). The most enriched GO categories in CK_L15 vs. DR_L15 up-regulated DEGs were “RNA modification”, “Cytoplasm”, “Response to heat”, “intracellular anatomical structure”, and “Response to temperature stimulus”. The most enriched GO categories in CK_R15 vs. DR_R15 up-regulated DEGs were “Response to heat”, “Response to abiotic stimulus”, “nucleolus”, “Response to temperature stimulus”, and “preribosome” (Figure 4A). In 45 days after transplanting, the most enriched GO categories in CK_L45 vs. DR_L45 up-regulated DEGs were “ribosome”, “structural constituent of ribosome”, “structural molecular activity”, “peptide metabolic process”, and “translation”. The most enriched GO categories in CK_R45 vs. DR_R45 up-regulated DEGs were “structural constituent of ribosome”, “ribosome”, “structural molecular activity”, “translation”, and “peptide biosynthetic process” (Figure 4A).

The most enriched GO categories in CK_L15 vs. DR_L15 down-regulated DEGs were “membrane”, “intrinsic component of membrane”, “integral component of membrane”, “cell periphery”, and “carbohydrate metabolic process”. The most enriched GO categories in CK_R15 vs. DR_R15 down-regulated DEGs were “cell periphery”, “extracellular region”, “intrinsic component of membrane”, “integral component of membrane”, and “plasma membrane”. The most enriched GO categories in CK_L45 vs. DR_L45 down-regulated DEGs were “carbohydrate metabolic process”, “cell periphery”, “membrane”, “intrinsic component of membrane”, and “integral component of membrane” (Figure 4B). The most enriched GO categories in CK_R45 vs. DR_R45 down-regulated DEGs were “peptidase regulator activity”, “peptidase inhibitor activity”, “endopeptidase regulator activity”, “endopeptidase inhibitor activity”, and “response to stress” (Figure 4B).

In both L15 and R15 tissues, up-regulated genes were specially enriched in the GO terms “GO:0009628 response to abiotic stimulus”, “GO:0009451 RNA modification”, “GO:0009408 response to heat”, “GO:0009622 response to temperature stimulus”, “GO:0006457 protein folding”, and “GO:0005622 intracellular anatomical structure”. In both L45 and R45 tissues, “GO:0003735 structural constituent of ribosome”, “GO:0005198 structural molecular activity”, and other protein biosynthesis-related GO terms are enriched.

The GO terms “GO:0016020 membrane”, “GO:0031224 intrinsic component of membrane”, and “GO:0005618 cell wall” were commonly enriched in L15, R15, and L45 stages and tissues. A total of 485 down-regulated DEGs in DR_R45 vs. CK_R45 showed distinct expression profiles in pathways such as “Ko:04141 Protein processing in endoplasmic reticulum”, “Ko:00196 Photosynthesis—antenna proteins”, and “Ko:04626 Plant-pathogen interaction”.

KEGG pathway enrichment analysis showed that “KO:03010 Ribosome”, “KO:03008 Ribosome biogenesis in eukaryotes”, “KO:04141 protein processing in endoplasmic reticulum”, “KO:02010 ABC transporter”, “KO:04120 Ubiquitin mediated proteolysis”, and “KO:04146 Peroxisome” are enriched.

As for KEGG pathway enrichment analysis of down-regulated DEGs, “KO:04626 plant-pathogen interaction”, “KO:00500 starch and sucrose metabolism”, “KO:00520 Amino sugar and nucleotide sugar metabolism”, “KO:00940 phenylpropanoid biosynthesis”, and “KO:00460 cyanoamino acid metabolism” are enriched (Figure 4D). These KEGG pathways are expected as drought inhibits sweet potato growth by suppressing starch and sucrose metabolism. The common down-regulated DEGs in all stages and tissues enriched in the KEGG pathway include “KO:00940 phenylpropanoid biosynthesis”, “KO:00520 Amino sugar and nucleotide sugar metabolism”, and “KO:00500 Starch and sucrose metabolism”. The commonly up-regulated DEGs include “KO:04120 Ubiquitin mediated proteolysis” and “Ko:03008 Ribosome biogenesis in eukaryotes” (Figure 4D).

### 3.5. Differentially Expressed Transcriptional Factors

Based on the Hammscan function [24], 301, 233, 473, and 40 DEGs were annotated as transcription factors in L15, L45, R15, and R45 in drought versus control, respectively (Table S4), with 389 up-regulated DEGs and 658 down-regulated DEGs in total. Among them, AP2-EREBP transcriptional factors (150 genes, 14.3%), MYB transcriptional factors (142 genes, 13.6%), bHLH transcriptional factors (104 genes, 9.9%), and WRKY transcriptional factors (56 genes, 5.3%) were the top four enriched TF families (Table S4).

It is reasonable that most of the ABI3VP1 transcriptional factors (with 22 members up-regulated and 10 down-regulated) and HSF transcriptional factors (with 16 members up-regulated and 8 down-regulated) are up-regulated, because the ABI3VP1 family members are involved in the ABA signaling pathway, and HSF family members have the function to activate the HSP proteins. A large proportion of TFs are down regulated, indicating that most gene expressions are repressed under drought stress. Some transcription factors are disproportionately down-regulated, such as the WRKY transcriptional factor (9 up-regulated and 47 down-regulated) and GRAS transcription factor members (6 up-regulated and 44 down-regulated), which may play a vital role in sweet potato root and stem growth and development.

### 3.6. Receptor-like Kinases Differentially Expressed in Response to Drought

**Figure 5:**
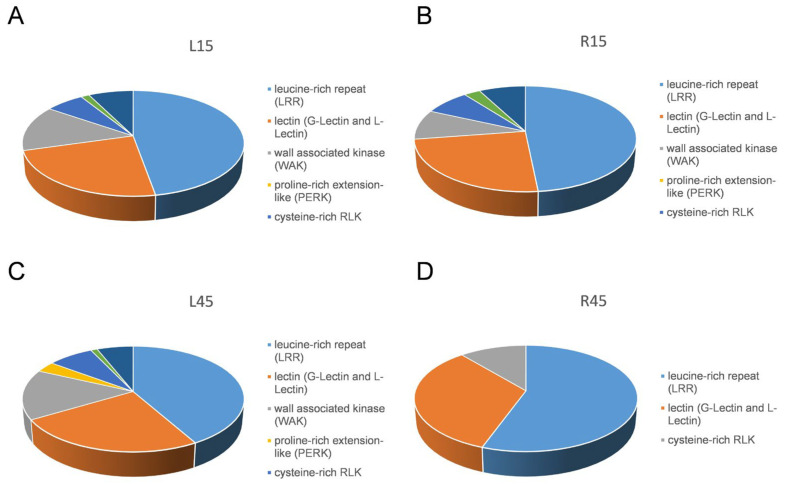
Receptor-like kinases enriched in (**A**) CK_L15 vs. DR_L15, (**B**) CK_L45 vs. DR_L45, (**C**) CK_R15 vs. DR_R15, and (**D**) CK_R45 vs. DR_R45.

On the basis of signature motifs in their extracellular domains, receptor-like kinases (RLKs) are categorized into 14 classes [25]. All transcription factors are listed in Supplemental Table S4. Using the term “kinase” as a keyword to search the DEGs list, a total of 316 (42 up-regulated and 274 down-regulated), 333 (54 up-regulated and 279 down-regulated), 290 (126 up-regulated and 164 down-regulated), and 16 (1 up-regulated and 15 down-regulated) receptor-like kinases are differentially expressed in the L15, R15, L45, and R45 stages in drought stress versus control, respectively (Figure 5). Among them, leucine-rich repeat (LRR) receptor kinases account for the largest proportion, followed by lectin (G-lectin and L-lectin) RLK, wall-associated kinase (WAK), cysteine-rich RLK, and leaf rust kinase-like RLKs (Figure 5).

## 4. Discussion

Sweet potato is one of the most important root crops cultivated worldwide [5]. It is a versatile crop that can be used as a nutritious food to alleviate vitamin A deficiency, and it can also be used as a raw material to produce starch and alcohol [5]. Due to the fluctuation of rainfall, especially rainfall shortage in the seedling stage, drought is an increasingly limiting factor for sweet potato yield.

Drought stress can greatly reduce the growth of sweet potato seedling leaves and roots, thus decreasing the seedling survival rate during the very early stage of transplanting. In the early stages of growth (0–60 d after transplanting), the number of leaves, leaf area, and stem length, as well as the formation and expansion of tuberous root, are also seriously affected [26]. The effect of drought on the fresh potato yield was more serious in the early stages than in the later stages [26].

The release of the sweet potato genome in 2017 greatly accelerated functional genome research [23]. However, the highly heterozygous hexaploid genome of *I. batatas* remains an obstacle. Transcriptome analysis makes it easy to uncover the defense mechanism against drought stress, which may further facilitate breeding for drought-tolerant sweet potato cultivars [11]. This study is the first to evaluate the drought-responsive genes and pathways in roots and leaves in both the seedling stage and tuberous expansion stage.

### 4.1. Pathways of Drought Tolerance in Different Tissues and Developmental Stages

Drought stress signal transduction consists of the ionic and osmotic homeostasis signaling pathway, detoxification (i.e., damage control and repair) response pathway, and pathways for growth regulation [3]. The number of DEGs is related to the drought tolerance degree. Previous studies showed that the extremely drought-tolerant sweet potato cultivar “xuzi-8” was almost unaffected by drought stress [12]. We can conclude that as sweet potatoes grow, their drought resistance increases, as evidenced by the significant decrease in the number of DEGs from 7119 in L15 to 5463 in L45.In roots, the number of DEGs decreased from 8811 in R15 to 930 in R45 (Table 2). As the sweet potato plant grows, root tissue becomes more tolerant to drought than leaf tissue, as indicated by the reduced number of DEGs from 8811 in R15 to 930 in R45, compared to 7119 in L15 to 5463 in L45 (Table 2).

From the perspective of the transplanting day, we can conclude that up-regulated DEGs in both L15 and R15 are assigned to “GO:0005622 intracellular anatomical structure”, “GO:0006457 protein folding”, “GO:0009628 response to abiotic stimulus”, “GO:0009451 RNA modification”, “GO:0009408 response to heat”, and “GO:0009266 response to temperature stimulus”. However, the situation in both L45 and R45 is different. Up-regulated DEGs are commonly assigned to “GO:0005840 ribosome”, “GO:0006421 translation”, “GO:0006518 peptide metabolic process”, “GO:0043228 non-membrane-bounded organelle”, and so on. All these GO terms are involved in protein biosynthesis processes (Figure 4).

The down-regulated DEGs in L15, L45, and R15 between drought and CK commonly were strongly enriched for membrane-related GO terms, including “GO:0031224 intrinsic component of membrane”, “GO:0016021 integral component of membrane”, “GO:0016020 membrane”, and “GO:0005886 plasma membrane” (Figure 4A). Under the GO term “GO:0016020 term membrane”, genes encoding ABC transporter, water channel protein aquaporin family members, ion transporters such as Na^+^ and K^+^ ion transporters and H^+^-ATPase, Ca^2+^ channel protein, and receptor-like kinases are all included. However, in the R45 stage, most enriched GO terms are assigned to “GO:0004866 endopeptidase inhibitor activity”, “GO:0016679 oxidoreductase activity, acting on diphenols and related substances as donors”, “GO:0006950, response to stress”, “GO:0030554 adenyl nucleotide”, “GO:0050896 response to stimulus”, and so on (Figure 4A). The most important gene families involved in drought stress in this study are discussed below.

### 4.2. ABA Biosynthesis and Signaling Pathway

The ABA signaling pathway is critical in regulating plant water balance and osmotic stress tolerance [3]. In this pathway, ABA binds to the soluble PYR/PYL/RCAR receptors, allowing the receptors to physically interact with and inhibit the activity of PP2C-A phosphatases, which in turn release SnRK2 kinases from their association with and inhibition by the PP2Cs [27].

DEGs related to ABA biosynthesis and signaling pathway in all four groups are assigned to “GO:0009737 response to abscisic acid”, “GO:0010427 abscisic acid binding”, “GO:0009738 abscisic acid-activated signaling pathway”, “GO:0071215 cellular response to abscisic acid stimulus”, “GO:0009787 regulation of abscisic acid-activated signaling pathway”, “GO:0009687 abscisic acid metabolic process”, and “GO:0009688 abscisic acid biosynthetic process”.

The ABA content expectedly increased in all four groups between drought treatment versus control (Figure 1). The *NCED* encodes 9-cis-epoxycarotenoid dioxygenase, a key ABA biosynthesis enzyme. Our results showed that *NCED* members (*g30636*, *g43412*, *g54338*, *g21310*) are expectedly upregulated in the L15 stage compared to control (Supplemental Table S1), consistent with the increase in ABA content. The above-mentioned genes can be assigned to the KEGG pathway “KO:00906 Carotenoid biosynthesis”. The abscisic acid receptor *PYL8* (*g26930*) was up-regulated as well, both in the L15 stage and L45 stage (Supplemental Table S1). The role of ABA in drought is diverse, encompassing at least two key aspects: water balance and cellular dehydration tolerance. The role in water balance is predominantly executed through the regulation of guard cells, whereas the latter facet pertains to triggering the expression of genes encoding dehydration-resistant proteins across virtually all cell types [3]. An important part of the transport of ABA to stomatal guard cells is ABA transporter proteins, such as *NITRATE TRANSPORTER 1* (*NRT1*). In the L45 stage, *g57285,* which was annotated as NRT1, was expectedly up-regulated (Table S2).

### 4.3. Plant–Pathogen Interaction

Drought stress affects plant pathogen defense as DEGs are enriched in “KO:04626 Plant-pathogen interaction”. In this pathway, the expression of a group of protein genes involved in signaling sensing like calcium-dependent protein kinases, LRR receptor-like kinases, and calmodulin are affected. “Disease resistance protein RPM1-like” takes the largest proportion. It is noticeable that most plant–pathogen interaction genes mentioned above are downregulated, and this indicates that drought stress may weaken the plant pathogen defense responses.

### 4.4. Osmotic Homeostasis

The genes related to osmotic homeostasis are mainly assigned to “GO:0022857 transmembrane transporter activity”. This GO term includes water channel protein aquaporins, osmotin-like protein TPM1, ion channels, ion transporters, NRT1 family members, sugar transporters, and sucrose transporters, among others.

### 4.5. Osmotin-like Protein TPM1

Osmotin is a cationic, secretory protein that targets the vacuole. It exerts antifungal activity and plays an important role in cold, salinity, and water deficiency tolerance [28]. Studies on tobacco cell cultures acclimated to 428 mM NaCl showed that osmotin accumulation represents 12% of the total cellular proteins [28]. Previous research on sweet potato drought tolerance showed that TPM-1 is sharply up-regulated after drought, heat, or combined stresses [29]. In our studies, TPM-1 genes were differentially expressed. Some members, like *g16028* in the L45 stage and *g16031* and *g15950* in the R15 stage, were up-regulated. There are still other members that were down regulated after drought.

### 4.6. Aquaporin

Aquaporin is a family of small proteins located in the membrane that mediate water, urea, glycerol, NH_3_/NH_4_^+^, and CO_2_ transmembrane transport [30]. Higher plant aquaporin members include TIP, NIP, SIP, and PIP subfamilies. TIP is localized in vacuolar membranes of seed, leaf, and root tissue. NIP is localized in root nodules. PIP is localized in the plasma membrane of roots, shoots, leaves, and florescence [30]. In our research, aquaporin *PIP1-2* (*g25287*) was consistently down-regulated in all four groups, aquaporin *TIP2-1* (*g55284*) was down-regulated in L15, and aquaporin *NIP1-1* (*g34699*) was down-regulated in R45. Fourteen aquaporin family members in “GO:0016020 membrane”, “GO:0005372 water transmembrane transporter activity”, and “GO:0015250 water channel activity” were downregulated. Of course, the downregulation of these proteins might be beneficial for water balance, but it also reduces the intake of small solutes and CO₂, thus reducing photosynthesis rate.

### 4.7. Damage Control and Repair

#### Heat Shock Proteins

In this research, heat shock proteins are significantly enriched throughout the seedling stage to tuberous expansion stage, especially in CK_L15-vs-DR_L15. These genes belonged to “GO:0009408 response to heat”, “GO:0005737 cytoplasm”. “GO:0009266 response to temperature stimulus”, and “GO:0006457 protein folding”. There are 12 genes (*g25552*, *g8564*, *g16833*, *g20696*, *g15521*, *g5701*, *g43750*, *g35180*, *g32248*, *g43749*, *g4871*, *g4824*, *g12491*, *g34885*) annotated as *HSP* members in the top 40 up-regulated genes screened by *p* value (Figure 3A and Supplemental Table S2). In CK_R15-vs-DR_R15, there are also seven genes (*g51792*, *g25552*, *g35180*, *g4824*, *g4897*, *g32241*, *g4871*) annotated as *HSP* in the up-regulated genes. Many of the morphological and phenotypic effects of heat stress can be explained by the aggregation of proteins and an imbalance of protein homeostasis in general [31]. Heat shock proteins are a group of small proteins that respond to heat and drought. The most conserved HSPs are molecular chaperones that prevent the formation of nonspecific protein aggregates and assist proteins in the acquisition of their native structures [31].

### 4.8. Sporamin

Sporamins are characterized as trypsin inhibitors and have been shown to have various antioxidant functions related to stress tolerance, such as DHA and MDA reductase activities [32]. This kind of protein is constitutively expressed in the tuberous root but not normally expressed in the stem or leaves [33]. We observed many sporamin genes including *g46653*, *g46661*, *g46666*, *g46667*, and *g46651* that were highly enriched in leaf tissues, both in L15 and L45 (Figure 3A,B). It has been stated that those proteins are expressed systemically in response to wounding and other abiotic stresses, Sporamin also possesses scavenging activity against 1,1-diphenyl-2-picrylhydrazyl (DPPH) and hydroxyl radicals as well as glutathione peroxidase-like activity [34], which may elucidate why those proteins are greatly up-regulated in non-root tissues during drought stress.

### 4.9. LEA Proteins

Late embryogenesis abundant (LEA) proteins are extremely hydrophilic proteins that can stabilize and protect other proteins and membranes during drying [35]. In both L15 and R15, the LEA protein dehydrin *DHN2* (*g17002*) is prominently upregulated between drought stress and the control. Another LEA protein gene, *g20722,* is constantly upregulated in all four stages and tissues. A previous study showed that LEA4-group genes from the resurrection plant *Boea hygrometrica, BhLEA1* and *BhLEA2*, confer dehydration tolerance in transgenic tobacco [36]. There are 14 LEA proteins/dehydrin genes enriched in L15 and R15 samples and only 3 LEA proteins/dehydrin genes in L45 samples (Supplemental Tables S2 and S3). This suggests that these genes play a more important role in the earlier stages of sweet potato.

### 4.10. Proteins Required for Detoxification

Drought stress causes reactive oxygen species generation and alters the cellular redox balance. On one hand, ROS acts as a signal to trigger drought response-related signaling pathways. The over-accumulation of ROS in cellular compartments results in increased plasma membrane permeability, decreased chlorophyll, and metabolic disorders.

Proteins in the ROS-scavenging system are necessary for cellular redox balance [37]. In this research, DEGs encoding proteins for detoxification, including glutathione S-transferase, ABC transporter, glutathione peroxidase, and superoxide dismutase (SOD), are assigned to the KEGG pathway “KO:04146 peroxisome”, “KO:00480 Glutathione metabolism”, and “KO:02010 ABC transporters”.

### 4.11. Lipid Metabolism

#### GDSL Esterase/Lipase

GDSL esterase/lipase proteins (GELPs) compose a family of enzymes identified by a unique “GDSL” amino acid sequence motif. GELPs can accept a broad range of substrates and are functionally diverse. Plant GELPs play roles mainly in modulating development, metabolism, response against pathogens and abiotic stresses, or cuticle biogenesis and degradation. In our study, a group of GDSL esterase/lipases were constantly downregulated in leaf tissues, for example, *g37717* in L15, *g54917*, *g57399*, *g11768*, *g57611*, and *g60792* in L45. It has been reported that overexpression of *GmGELP28* and *ZmAchE* increased drought and heat tolerance, and GELP mutants with cutinase or transferase activity caused rapid water loss [38].

### 4.12. Growth Inhibition

The growth of sweet potato is severely inhibited under drought stress. The biomass of both sweet potato roots and shoots declined sharply under drought conditions (Supplemental Figure S2). Many genes and pathways are involved in growth inhibition under drought stress. The affected genes are mainly assigned to the following pathways: “KO:04075 Plant hormone signal transduction”, “KO:00908 Zeatin biosynthesis”, “KO:00905 Brassinosteroid biosynthesis”, “KO:00500 Starch and sucrose metabolism”, “KO:00710 Carbon fixation in photosynthetic organisms”, and “Ko:00195 Photosynthesis”.

As we can see in our research, almost all kinds of plant hormone-related genes are affected in plant hormone signal transduction pathways. Among them, the auxin-induced genes *IAAs* and *small auxin up RNA SAUR* account for more than half (Supplemental Tables S2 and S3). A group of zeatin O-xylosyltransferase-like protein genes which play a key role in cytokinin biosynthesis are downregulated(Supplemental Table S3). The GA20 oxidase GA20OX1-encoding gene (*g20539*) and others involved in GA and auxin biosynthesis and their signaling pathways, which play important roles in stem elongation, are also down-regulated (Figure 3A).

Drought stress will rebalance normal growth and drought-induced response metabolism. Both in L45 and R45, between drought treatment and control, the ribonucleoprotein complex, including a group of 30S, 40S, and 60S ribosome proteins, was enriched in the pathway “KO:03010 Ribosome”. It has been reported that ribosomal components increased in *Arabidopsis thaliana* [39] and *Gossypium hirsutum* [40] under salt stress. Like salt stress, drought stress appears to cause a similar effect. And total protein contents increased after drought in all stages and tissues, as expected (Figure 1).

## 5. Conclusions

In conclusion, this research found that drought stress can seriously inhibit the growth of sweet potato. The concentration of ABA increased significantly after drought stress. In addition, drought-responsive genes in the roots and leaves of sweet potato at different transplanting stages have distinct expression profiles. Compared with leaf tissue, the number of differentially expressed genes in root tissue was smaller, indicating that roots were less sensitive to drought stress.More differentially expressed genes at the seedling stage indicated that seedlings were more sensitive to drought stress. Drought-response genes were mainly involved in ABA signaling pathways, osmotic homeostasis, damage control and repair, detoxification, lipid metabolism, and growth regulation signaling pathways. These results provide new insights and candidate genes for further study of drought tolerance mechanisms of sweet potato.

## Figures and Tables

**Table 1:** Overview of transcript sequencing in two developmental stages.

Sample	Clean Reads Pairs	Clean Base (bp)	Length	Q20 (%)	Q30 (%)	GC (%)
CK_L15-1	26,937,669	8,081,300,700	150; 150	98.19; 97.09	94.74; 91.89	47.74; 47.70
CK_L15-2	33,997,884	10,199,365,200	150; 150	98.19; 97.66	94.74; 93.26	48.61; 48.60
CK_L15-3	30,294,698	9,088,409,400	150; 150	98.19; 98.01	94.73; 94.09	48.67; 48.66
CK_L45-1	27,583,944	8,275,183,200	150; 150	98.17; 97.74	94.70; 93.42	47.73; 47.72
CK_L45-2	26,057,986	7,817,395,800	150; 150	98.12; 97.31	94.54; 92.39	46.80; 46.77
CK_L45-3	29,755,864	8,926,759,200	150; 150	98.11; 97.43	94.52; 92.70	47.48; 47.47
CK_R15-1	28,985,437	8,695,631,100	150; 150	98.19; 98.05	94.72; 94.14	46.18; 46.16
CK_R15-2	27,361,580	8,208,474,000	150; 150	98.15; 97.31	94.64; 92.42	46.43; 46.41
CK_R15-3	24,953,901	7,486,170,300	150; 150	98.15; 97.82	94.65; 93.61	46.09; 46.05
CK_R45-1	29,480,386	8,844,115,800	150; 150	98.18; 97.78	94.69; 93.54	48.82; 48.77
CK_R45-2	25,654,529	7,696,358,700	150; 150	98.17; 97.87	94.65; 93.75	48.71; 48.68
CK_R45-3	27,392,172	8,217,651,600	150; 150	98.19; 97.21	94.72; 92.21	49.10; 49.05
DR_L15-1	29,058,008	8,717,402,400	150; 150	98.16; 97.45	94.65; 92.74	47.22; 47.19
DR_L15-2	26,690,924	8,007,277,200	150; 150	98.16; 97.70	94.64; 93.31	47.38; 47.34
DR_L15-3	27,290,514	8,187,154,200	150; 150	98.16; 97.23	94.67; 92.27	47.63; 47.61
DR_L45-1	26,082,650	7,824,795,000	150; 150	98.13; 97.59	94.58; 93.05	47.26; 47.22
DR_L45-2	24,928,074	7,478,422,200	150; 150	98.12; 97.70	94.56; 93.34	47.10; 47.06
DR_L45-3	26,047,394	7,814,218,200	150; 150	98.14; 98.12	94.65; 94.37	47.33; 47.30
DR_R15-1	27,467,948	8,240,384,400	150; 150	98.18; 97.81	94.70; 93.57	46.40; 46.36
DR_R15-2	28,567,133	8,570,139,900	150; 150	98.11; 97.83	94.54; 93.64	46.29; 46.27
DR_R15-3	31,338,669	9,401,600,700	150; 150	98.17; 97.87	94.67; 93.74	46.12; 46.10
DR_R45-1	28,006,857	8,402,057,100	150; 150	98.17; 97.86	94.69; 93.74	48.93; 48.87
DR_R45-2	26,002,898	7,800,869,400	150; 150	98.19; 97.30	94.75; 92.42	49.24; 49.22
DR_R45-3	23,126,099	6,937,829,700	150; 150	98.21; 97.93	94.77; 93.88	48.55; 48.53

In each stage (tissue), there are three biological replicates from every single individual plant. Each sample represents a biological replicate individually to form a unique library separately. CK: control; DR: drought stress. L15 and L45 indicate leaves from sweet potato 15 and 45 days after transplanting. R15 and R45 indicate roots from sweet potato 15 and 45 days after transplanting.

**Table 2:** Overview of DEGs in L15, L45, R15 and R45.

Group	Total	Up	Down
CK_L15-vs-DR_L15	7119	2906	4213
CK_R15-vs-DR_R15	8811	2996	5815
CK_L45-vs-DR_L45	5463	1744	3719
CK_R45-vs-DR_R45	930	445	485

CK: control; DR: drought stress. L15 and L45 indicate leaves from sweet potato 15 and 45 days after transplanting. R15 and R45 indicate roots from sweet potato 15 and 45 days after transplanting.

## Data Availability

The Illumina NGS reads generated in this study are available from the GSA at CNCB (https://ngdc.cncb.ac.cn/gsa/s/d0M11JSH, accessed on 30 April 2024) under the accession number CRA016124.

## Supplementary Materials

The following supporting information can be downloaded at https://www.mdpi.com/article/10.3390/genes15070948/s1, Figure S1: gene correlation; Figure S2: trait data; Table S1: Mapped_ratio; Table S2: Up-regulated DEGs; Table S3: Down-regulated DEGs; Table S4: TF_coding_genes.

## References

[1] Gupta A., Rico-Medina A., Cano-Delgado A.I. (2020). The physiology of plant responses to drought. Science.

[2] Berger J., Palta J., Vadez V. (2016). Review: An integrated framework for crop adaptation to dry environments: Responses to transient and terminal drought. Plant Sci..

[3] Zhu J.-K. (2002). Salt and Drought Stress Signal Transduction in Plants. Annu. Rev. Plant Biol..

[4] Zhu J.-K. (2016). Abiotic Stress Signaling and Responses in Plants. Cell.

[5] Amagloh F.C., Yada B., Tumuhimbise G.A., Amagloh F.K., Kaaya A.N. (2021). The Potential of Sweetpotato as a Functional Food in Sub-Saharan Africa and Its Implications for Health: A Review. Molecules.

[6] Chen L.L., Wang L., Wu Y.J., Chen X.L., Kong X., Gong J.Y., Wang J.F., Yi Y., Xiong H. (2023). Molecular and Microecological Mechanisms of Plant Responses to Drought Stress. Mol. Plant Breed..

[7] Yan H., Zhang Y., Ahmad M.Q., Liu Y., Kou M., Ma M., Li C., Arisha M.H., Tang W., Wang X. (2022). Comparative Analysis of Anthocyanin Compositions and Starch Physiochemical Properties of Purple-Fleshed Sweetpotato “Xuzishu8” in Desert Regions of China. Front. Plant Sci..

[8] De Rossi S., Di Marco G., Bruno L., Gismondi A., Canini A. (2021). Investigating the Drought and Salinity Effect on the Redox Components of *Sulla Coronaria* (L.) Medik. Antioxidants.

[9] Rai G.K., Parveen A., Jamwal G., Basu U., Kumar R.R., Rai P.K., Sharma J.P., Alalawy A.I., Al-Duais M.A., Hossain M.A. (2021). Leaf Proteome Response to Drought Stress and Antioxidant Potential in Tomato (*Solanum lycopersicum* L.). Atmosphere.

[10] Lau K.H., Del Rosario Herrera M., Crisovan E., Wu S., Fei Z., Khan M.A., Buell C.R., Gemenet D.C. (2018). Transcriptomic analysis of sweet potato under dehydration stress identifies candidate genes for drought tolerance. Plant Direct.

[11] Arisha M.H., Aboelnasr H., Ahmad M.Q., Liu Y., Tang W., Gao R., Yan H., Kou M., Wang X., Zhang Y. (2020). Transcriptome sequencing and whole genome expression profiling of hexaploid sweetpotato under salt stress. BMC Genom..

[12] Liu E., Xu L., Luo Z., Li Z., Zhou G., Gao H., Fang F., Tang J., Zhao Y., Zhou Z. (2023). Transcriptomic analysis reveals mechanisms for the different drought tolerance of sweet potatoes. Front. Plant Sci..

[13] Xu Y., Chen Y., Fu Z.G. (2004). Advance of research on drought resistant physiology and cultivation techniques of sweet potato. Agric. Res. Arid. Areas.

[14] Zhang L., Gao W., Cao Z., He L.S., Tan G.Y., Wang B.M. (2014). Immunolocalization and Quantitation of ABA and IAA in the Organs of Wheat (*Triticum aestivum* L.) Under Drought Stress. Sci. Agric. Sin..

[15] Chen Y., Chen Y., Shi C., Huang Z., Zhang Y., Li S., Li Y., Ye J., Yu C., Li Z. (2018). SOAPnuke: A MapReduce acceleration-supported software for integrated quality control and preprocessing of high-throughput sequencing data. Gigascience.

[16] Kim D., Paggi J.M., Park C., Bennett C., Salzberg S.L. (2019). Graph-based genome alignment and genotyping with HISAT2 and HISAT-genotype. Nat. Biotechnol..

[17] Langmead B., Salzberg S.L. (2012). Fast gapped-read alignment with Bowtie 2. Nat. Methods.

[18] Li B., Dewey C.N. (2011). RSEM: Accurate transcript quantification from RNA-Seq data with or without a reference genome. BMC Bioinform..

[19] Love M.I., Huber W., Anders S. (2014). Moderated estimation of fold change and dispersion for RNA-seq data with DESeq2. Genome Biol..

[20] Ashburner M., Ball C.A., Blake J.A., Botstein D., Butler H., Cherry J.M., Davis A.P., Dolinski K., Dwight S.S., Eppig J.T. (2000). Gene ontology: Tool for the unification of biology. The Gene Ontology Consortium. Nat. Genet..

[21] Kanehisa M., Goto S., Kawashima S., Okuno Y., Hattori M. (2004). The KEGG resource for deciphering the genome. Nucleic Acids Res..

[22] Yan M., Nie H., Wang Y., Wang X., Jarret R., Zhao J., Wang H., Yang J. (2022). Exploring and exploiting genetics and genomics for sweetpotato improvement: Status and perspectives. Plant Commun..

[23] Yang J., Moeinzadeh M.H., Kuhl H., Helmuth J., Xiao P., Haas S., Liu G., Zheng J., Sun Z., Fan W. (2017). Haplotype-resolved sweet potato genome traces back its hexaploidization history. Nat. Plants.

[24] Potter S.C., Luciani A., Eddy S.R., Park Y., Lopez R., Finn R.D. (2018). HMMER web server: 2018 update. Nucleic Acids Res..

[25] Gandhi A., Oelmüller R. (2023). Emerging Roles of Receptor-like Protein Kinases in Plant Response to Abiotic Stresses. Int. J. Mol. Sci..

[26] Xiao L.Z. (1995). Influence of soil aridity on the growth, development and yield of sweet potato (*Ipomoea batatas* L.). Acta Agric. Boreali-Sin..

[27] Zhang H., Zhu J., Gong Z., Zhu J.-K. (2021). Abiotic stress responses in plants. Nat. Rev. Genet..

[28] Viktorova J., Krasny L., Kamlar M., Novakova M., Mackova M., Macek T. (2012). Osmotin, a pathogenesis-related protein. Curr. Protein Pept. Sci..

[29] Tang W., Arisha M.H., Zhang Z., Yan H., Kou M., Song W., Li C., Gao R., Ma M., Wang X. (2022). Comparative transcriptomic and proteomic analysis reveals common molecular factors responsive to heat and drought stresses in sweetpotaoto (*Ipomoea batatas*). Front. Plant Sci..

[30] Kaldenhoff R., Fischer M. (2006). Aquaporins in plants. Acta Physiol..

[31] Richter K., Haslbeck M., Buchner J. (2010). The Heat Shock Response: Life on the Verge of Death. Mol. Cell.

[32] Yeh K.W., Chen J.C., Lin M.I., Chen Y.M., Lin C.Y. (1997). Functional activity of sporamin from sweet potato (*Ipomoea batatas* Lam.): A tuber storage protein with trypsin inhibitory activity. Plant Mol. Biol..

[33] Senthilkumar R., Yeh K.-W. (2012). Multiple biological functions of sporamin related to stress tolerance in sweet potato (*Ipomoea batatas* Lam). Biotechnol. Adv..

[34] Hou W.C., Chen Y.C., Chen H.J., Lin Y.H., Yang L.L., Lee M.H. (2001). Antioxidant activities of trypsin inhibitor, a 33 KDa root storage protein of sweet potato (*Ipomoea batatas* (L.) Lam cv. Tainong 57). J. Agric. Food Chem..

[35] Hand S.C., Menze M.A., Toner M., Boswell L., Moore D. (2011). LEA Proteins During Water Stress: Not Just for Plants Anymore. Annu. Rev. Physiol..

[36] Liu X., Wang Z., Wang L., Wu R., Phillips J., Deng X. (2009). LEA 4 group genes from the resurrection plant *Boea hygrometrica* confer dehydration tolerance in transgenic tobacco. Plant Sci..

[37] Athar H.-R., Zulfiqar F., Moosa A., Ashraf M., Zafar Z.U., Zhang L., Ahmed N., Kalaji H.M., Nafees M., Hossain M.A. (2022). Salt stress proteins in plants: An overview. Front. Plant Sci..

[38] Aparato V.P.M., Suh D.Y. (2022). The functions and applications of GDSL esterase/lipase proteins in agriculture: A review. JSFA Rep..

[39] Huang K.C., Lin W.C., Cheng W.H. (2018). Salt hypersensitive mutant 9, a nucleolar APUM23 protein, is essential for salt sensitivity in association with the ABA signaling pathway in Arabidopsis. BMC Plant Biol..

[40] Li W., Zhao F., Fang W., Xie D., Hou J., Yang X., Zhao Y., Tang Z., Nie L., Lv S. (2015). Identification of early salt stress responsive proteins in seedling roots of upland cotton (*Gossypium hirsutum* L.) employing iTRAQ-based proteomic technique. Front. Plant Sci..

