# Supplementary figures and images for: Transcriptome Analysis Reveals Genes and Pathways Associated with Drought Tolerance of Early Stages in Sweet Potato (Ipomoea batatas (L.) Lam.)
Source: Genes (Basel). 2024 Jul 19;15(7):948. doi: 10.3390/genes15070948 (PMC11276569; doi:10.3390/genes15070948)

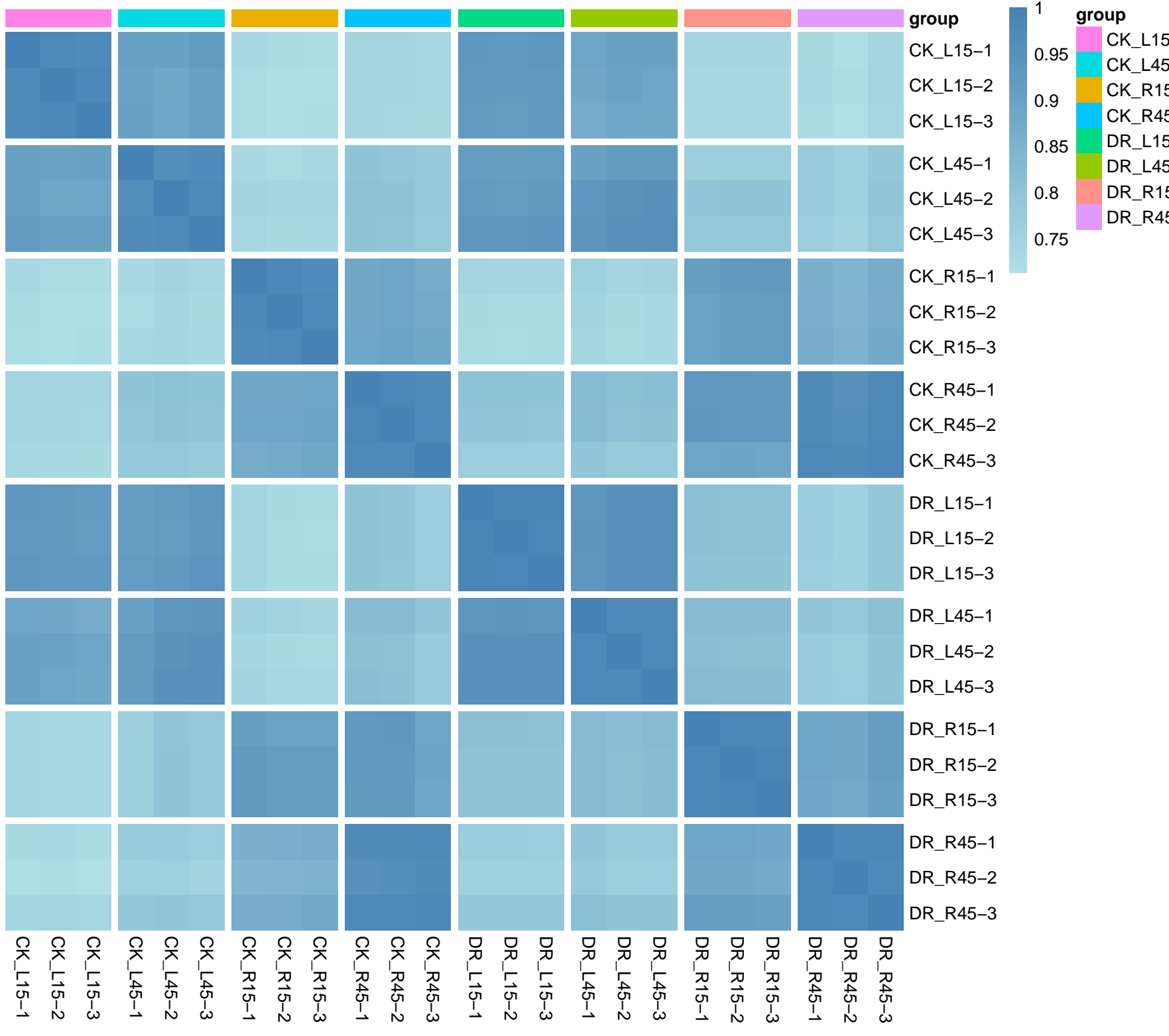

Supplement: Supplementary file 1 [file genes-15-00948-s001.zip › Supplemental figure S1 gene.correlation.pdf]

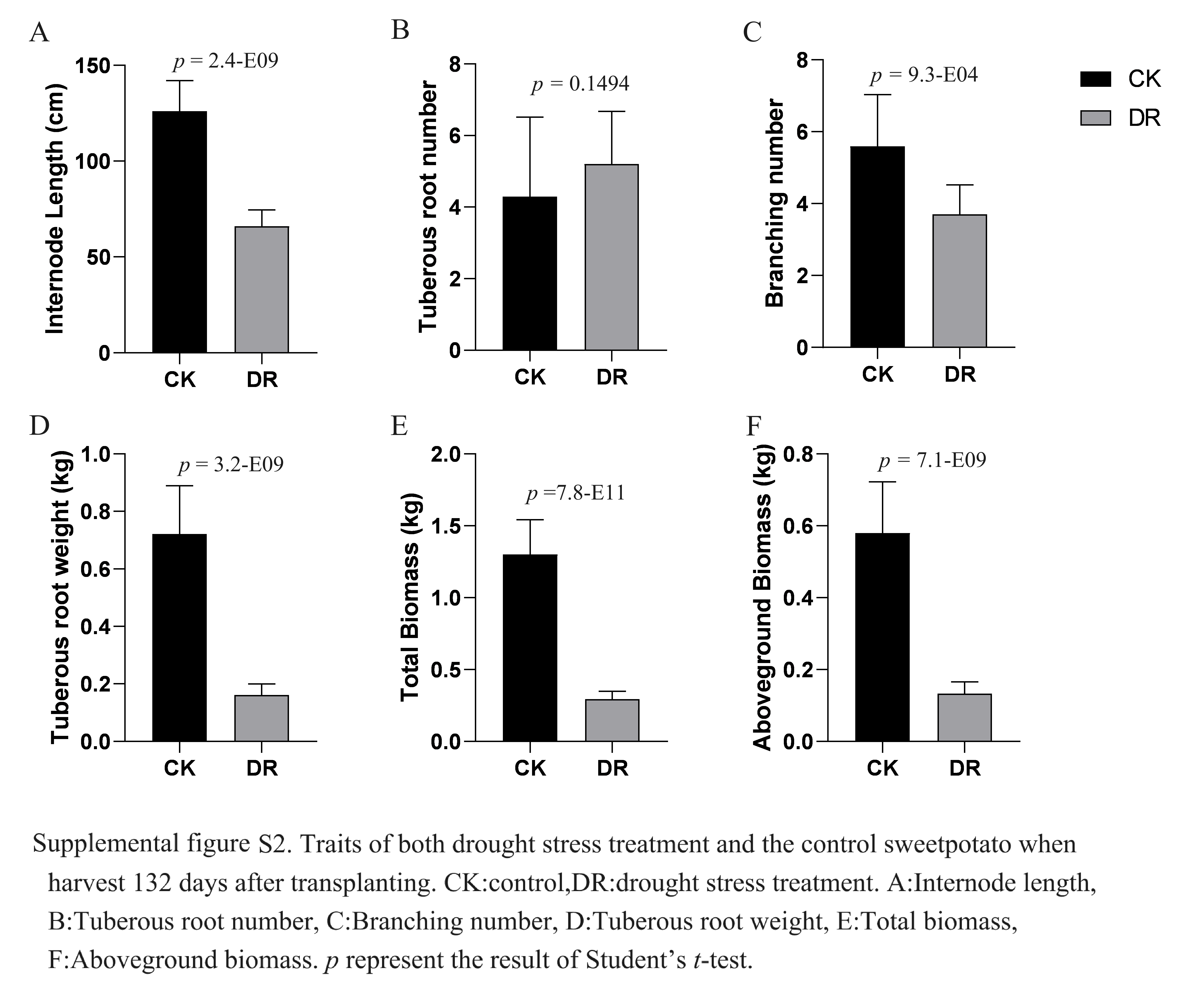

Supplement: Supplementary file 1 [file genes-15-00948-s001.zip › Supplemental figure S2 trait data.png]
